# Evaluation of P22 ELISA for the Detection of *Mycobacterium bovis*-Specific Antibody in the Oral Fluid of Goats

**DOI:** 10.3389/fvets.2021.674636

**Published:** 2021-08-11

**Authors:** Javier Ortega, José A. Infantes-Lorenzo, Javier Bezos, Álvaro Roy, Lucia de Juan, Beatriz Romero, Inmaculada Moreno, Alberto Gómez-Buendía, Irene Agulló-Ros, Lucas Domínguez, Mercedes Domínguez

**Affiliations:** ^1^VISAVET Health Surveillance Centre, Complutense University of Madrid, Madrid, Spain; ^2^Departamento de Sanidad Animal, Facultad de Veterinaria, Universidad Complutense de Madrid, Madrid, Spain; ^3^Unidad de Inmunología Microbiana, Centro Nacional de Microbiología, Instituto de Investigación Carlos III, Madrid, Spain; ^4^Grupo de Investigación en Sanidad Animal y Zoonosis, Departamento de Anatomía y Anatomía Patológica Comparadas, Facultad de Veterinaria, Universidad de Córdoba, Córdoba, Spain

**Keywords:** diagnosis, goat, tuberculosis, oral fluid, P22 ELISA

## Abstract

The ante-mortem diagnosis of tuberculosis (TB) in ruminants is based mainly on the intradermal tuberculin test and the IFN-γ assay. Antibody (Ab)-based tests have emerged as potential tools for the detection of TB infected animals using serum, plasma, or even milk samples. Oral fluids have also been evaluated as alternative samples with which to detect specific Abs against *Mycobacterium bovis* in pigs or wild boars, but not in ruminants. The objective of this study was, therefore, to evaluate the performance of an in house-ELISA for TB diagnosis (P22 ELISA) in goats as an experimental model for the diagnosis of TB using oral fluid samples. Oral fluid samples from 64 goats from a TB-infected herd (*n* = 197) and all the animals from a TB-free herd (*n* = 113) were analyzed using the P22 ELISA. The estimated sensitivity (Se) and specificity (Sp) were 34.4% (95% CI: 22.4–45.6) and 100% (95% CI: 97.4–100), respectively. The optimal cut-off point was set at 100% according to the ROC analysis. Those animals with a higher level of Abs in their oral fluid attained a higher lesion score (*p* = 0.018). In fact, when taking into account only the setting of the animals with severe lesions (*n* = 16), the ELISA showed a Se of 75% (95% CI: 53.7–96.2). Results of the present study suggest that the P22 ELISA is highly specific but has a limited value detecting infected animals in oral fluid samples. Nevertheless, its performance is significantly higher in the presence of severe lesions.

## Introduction

Animal tuberculosis (TB) is a zoonotic infection that is caused mainly by *Mycobacterium bovis* and, more rarely, by other members of the *Mycobacterium tuberculosis* complex (MTBC) (1). The control programmes carried out for ruminants such as cattle and goats are based principally on a test and cull strategy using the single and comparative intradermal tuberculin (SIT and CIT) tests, both of which are based on cell-mediated immune response (2). The interferon-gamma release assay (IGRA), which is an official ancillary diagnostic test for bovine TB, is used to maximize the detection of infected animals and is also based on the cellular immune response (3).

Serological tests have, in recent years, emerged as a potential ancillary test for livestock, and may even be a first option for wildlife owing to their advantages when compared to cell-based tests (4, 5). Moreover, serological tests could be a valuable diagnostic tool in the form of screening tests with which to detect TB at the herd level and have been shown to maximize the detection of TB infected ruminants when they are used in combination with cellular-based tests (6–8). An ELISA based on the recently developed P22 protein complex (P22 ELISA) has shown high performance in terms of sensitivity (Se) and specificity (Sp) in ruminants (4, 6, 8, 9).

Non-invasive and easy-to-collect samples other than serum and plasma have also been evaluated for the detection of specific Abs against several diseases including TB, one of which is oral fluids (10–13). Previous studies have evaluated the performance of the ELISA by comparing milk samples with those of serum for TB diagnosis, and have obtained a similar Se and Sp (14, 15). The use of milk samples is, however, restricted to dairy animals. Oral fluid, which is a biological fluid, can, meanwhile, allow the routine monitoring of animals' health status owing to the minimally invasive and non-stressful method employed to collect it, which can be performed by personnel with minimal training. Moreover, unlike the case of milk samples, it is not necessary for the animal to be lactating and allows males and those animals that do not produce milk (kids, females not lactating or meat herds) to be sampled (11) Oral fluid samples have additionally been proposed as an alternative biological specimen by which to detect specific Abs against *M. bovis* in wild boar, attaining a Se and a Sp of 67.3 and 100%, respectively (13). In fact, oral fluid samples have been used in the fight against other swine diseases, such as classical swine fever, influenza, or porcine reproductive and respiratory syndrome (PRRS) (16–20), thus suggesting that oral fluids are valuable samples for the surveillance and control of TB and other diseases in suids through the use of Ab-based diagnostic platforms (21).

Despite their use in swine populations, few studies have evaluated serological tests by employing oral fluid samples in ruminants in order to detect antibodies against foot and mouth disease virus (FMDV) or Schmallenberg virus (SBV) in bovines (22, 23). With regard to TB, no studies using oral fluid samples for the antibody-based diagnosis of TB in domestic ruminants have, to the best of the authors' knowledge, been published previously.

Given the usefulness of oral fluid samples in other species, we have adapted a P22 ELISA in order to analyze oral fluid samples in goats. The main objective of the present study was to evaluate, for the first time, the usefulness of oral fluid samples as regards detecting specific antibodies against *M. bovis* in ruminants.

## Materials and Methods

### Study Design and Herds of Study

The study was performed with two herds of Guadarrama-breed goats located in central Spain, one of which was *M. bovis*-infected (range: 1–7 year old animals) and one of which was TB-free (range: 1–6 year old animals), which were used for Se and Sp estimations, respectively. The Se was evaluated in a herd (*n* = 197) in which *M. bovis* SB0121 was isolated. This herd was subjected to a SIT test, a CIT test and an IGRA, which showed an apparent prevalence of 87.5, 67.7, and 53.8%, respectively. Owing to the high proportion of reactors, all the animals were slaughtered and subjected to *post-mortem* analysis. The presence of TB-compatible lesions was evaluated during slaughtering, and tissue samples were collected for the bacteriological culture and isolation of bacteria in the laboratory. Culture positive (*M. bovis* SB0121 was isolated) animals and those with TB compatible lesions (*n* = 64) were included in the study in order to evaluate the Se of the P22 ELISA in oral fluid samples. The Sp was evaluated in a TB-free herd (*n* = 113), based on its history of TB-free status and the negative results obtained from the animals' SIT, CIT, and serological tests (P22 ELISA) in the last three testing events in the last 2 years. The Gudair vaccine (CZ Vaccines, Porriño, Spain) against *M. avium* subsp. *paratuberculosis* (MAP) had been administered to the animals in both herds at the age of 6 months as part of their vaccination programmes.

The animals included in the study were subjected to SIT and CIT tests and IGRA. Serum and oral fluid samples were collected before the intradermal test and analyzed using a P22 ELISA. The results obtained were compared with the *post-mortem* analysis.

The animals in the present study were not considered as experimental animals. All handling and sampling procedures were performed in compliance with Spanish legislation (Royal Decree 720.7/2011).

### Serum and Oral Fluid Sample Collection

Blood samples were collected from the jugular vein by means of venipuncture, using plastic serum tubes (BD Vacutainer Becton, Dickinson and Company, Franklin Lakes, USA). The samples were stored at room temperature for 24 h and then centrifuged for 15 min at 650 g, after which sera were stored at −20°C until assaying. Oral fluid samples were obtained from the same goats by dry swabbing the animals' mouths, which were cleaned beforehand. In the laboratory, the dry swabs were introduced into 2 ml tubes with a medium composed of 4 μl of azidiol (Panreac, Spain) and 1 ml of phosphate buffered saline (PBS). These dry swabs were conserved at 4°C for 8 h, after which the samples were centrifuged at 13,000 g for 5 min, and an aliquot of 500 μl of the supernatant was obtained and stored at −20°C until the assay test.

### P22 ELISA

Serum samples were analyzed by employing a P22 ELISA, as previously described by Infantes-Lorenzo et al. (4). The protocol was adapted to oral fluid samples as follows: the optimal dilution of oral fluid was determined by evaluating the reactivity of samples diluted from 1:2 to 1:128, and a dilution of 1:2 was eventually chosen. One hundred microliters of detection antibody at 1:2,000 were added, and the plates were incubated at room temperature for 30 min. As before, the secondary antibody [Rabbit anti sheep IgG(H/L)-HRP] (Southern Biotech, USA) was titrated from 1:500 to 1:8,000 in order to choose the optimal dilution.

The results were expressed as a P22 ELISA percentage (E%), which was calculated using the following formula:

$$\begin{array}{l} \text{E}\%=\left[\text{mean sample OD}/\left(2\times \text{mean of negative control OD}\right)\right]\\ \;\times 100 \end{array}$$

In the case of the serum samples, E% values of 100 or higher were considered positive, as described elsewhere (4), while in that of oral fluid samples, a cut-off was calculated using a receiver operating characteristic (ROC) analysis, and E% values of 100 or higher were considered positive.

### Intradermal Tuberculin Tests

The SIT and CIT tests were carried out by means of the intradermal inoculation of 0.1 ml of bovine and avian PPDs (CZ Vaccines, Porriño, Spain) in the right and left site of the cervical region, respectively, using a Dermojet syringe (Akra Dermojet, Pau, France). All tests were performed according to Council Directive 64/432/EEC and Royal Decree RD2611/1996, and the reactions were interpreted as previously described (24).

### Interferon-Gamma Release Assay

Blood samples were collected from the jugular vein using evacuated tubes (BD Vacutainer Becton, Dickinson and Company, Franklin Lakes, USA) with heparin in order to detect IFN-γ production. The blood samples were then processed as previously described (25). The blood was incubated at 37°C in a humidified atmosphere in the presence of antigens (PPD-B and PPD-A) for 18–20 h. The samples were then centrifuged at 2,500 rpm for 15 min, and the supernatant was collected. Interferon-gamma release was measured using a commercial IGRA designed for goats (Bovigam TB kit, Thermo Fisher Scientific, Waltham, USA), according to manufacturer's instructions, and the results were interpreted as described elsewhere (26).

### Post-mortem Analysis

TB-compatible gross lesions (TBL) include nodular off-white lesions containing caseous material, which may be mineralized in the center and encapsulated by fibrous tissue (27, 28). The inspection and semi-quantitative scoring of the TBL present in the lung lobes and lymph nodes (LNs) of culled animals were carried out on the basis of a previous lesion valuation model proposed by Vordermeier et al. (29), with some modifications. This scoring system relies on the size and number of lesions, in addition to the percentage of the organ affected, as follows: 0, no visible lesions; 1, one small lesion apparent on slicing; 2, <5 lesions of <10 mm in diameter; 3, more than five lesions of <10 mm in diameter or one lesion >10–30 mm in diameter and/or <50% of the organ affected; 4, more than one lesion >30 mm in diameter and/or >50% of the organ affected; and 5, coalescing lesions and >70% of the organ affected. In the lungs, one extra point was awarded to animals that had pleural adhesions. All the lung lobes (left apical, left diaphragmatic, right apical, right cardiac, right accessory, and right diaphragmatic) were examined individually and the scores of these lobes were added up in order to calculate the total lung score. The head LN (retropharyngeal) and pulmonary LNs (tracheobronchial and mediastinal) were likewise examined individually, and the scores of the different LNs were added up in order to calculate the total score.

Tissue samples from the lungs and the retropharyngeal, tracheobronchial, and mediastinal LNs from 64 animals with TB lesions were used for bacteriological culture in Löwenstein-Jensen with sodium pyruvate medium (Difco, Spain), as described previously (15). Animals with TB-compatible lesions in the lungs or in the different LNs analyzed in the present study were considered as TB positive. A comparison between the P22 ELISA results and the lesion score obtained for the lungs and/or head and pulmonary LNs was then carried out.

### Statistical Analysis

Wilson's 95% confidence intervals (95% CI) were calculated for the percentage of positive reactors to the different tests. ROC analysis was performed to define the optimal cut-off value (Supplementary Material). Quantitative values, such as E% in oral fluid samples with regard to the TBL score, were attained for the two different herds and were compared using the Mann–Whitney *U*-test. Moreover, the variation of quantitative values of the diagnostic techniques regarding to the TBL score was calculated using an *R*-squared (*R*^2^) and interpretated as follows: 0.00–0.25 poor, 0.26–0.50 fair, 0.51–0.75 moderate, and 0.76–1.00 substantial. All the statistical tests were carried out using SPSS Statistics 25 (IBM, New York, NY, USA), and interpreted by considering a *p*-value of 0.05 in order to determine statistical significance.

## Results

With regard to the P22 ELISA carried out using oral fluid samples, a ROC curve was calculated using samples from TB-infected (*n* = 64) and TB-free (*n* = 113) animals. The optimal cut-off point was set at 100%, at which the highest Se and Sp were observed. A higher or lower cut-off point caused a loss of Se with a constant Sp, or vice versa, and it was for this reason that an E% of 100 was chosen as the cut-off point.

**Figure 1:**
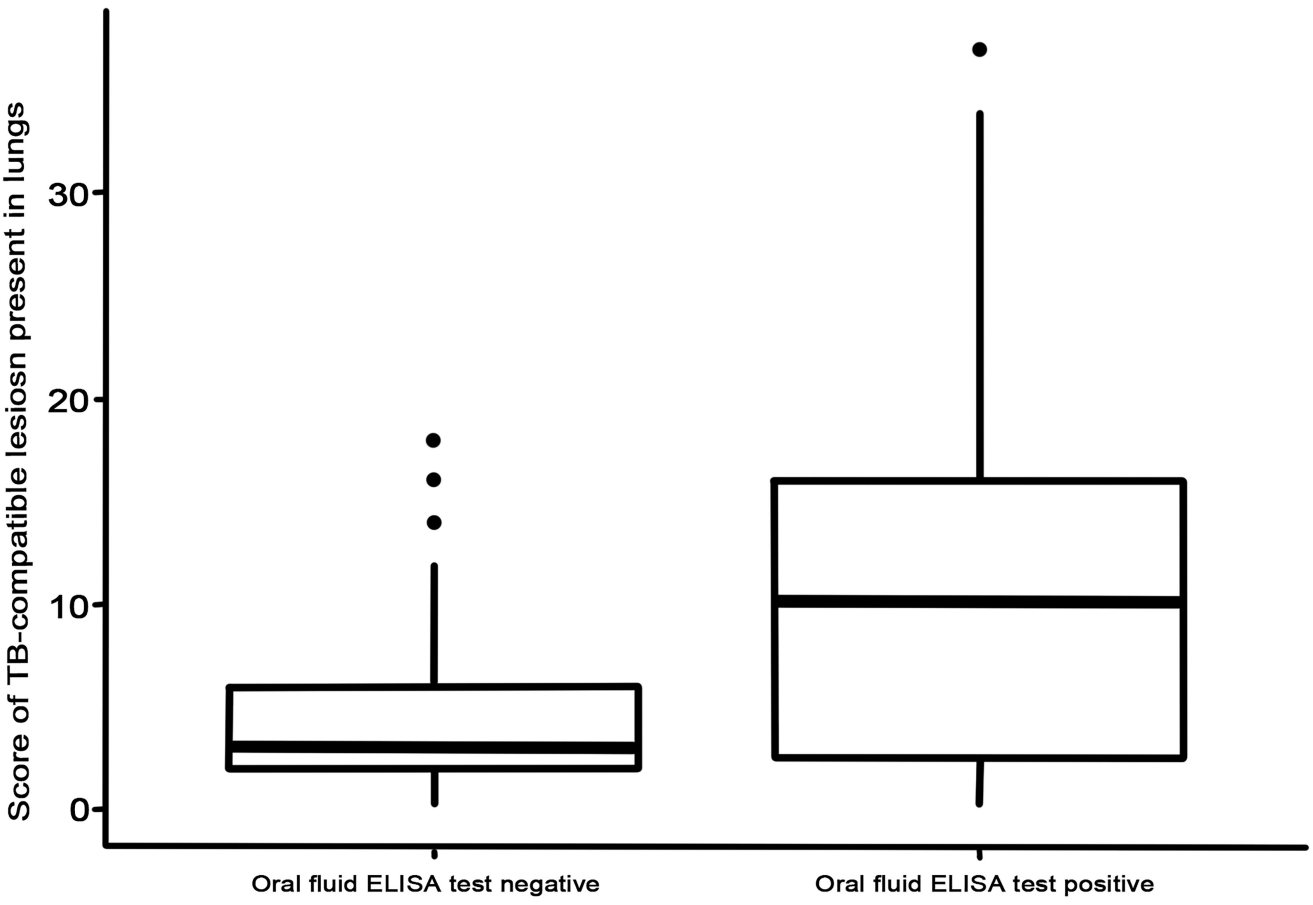
Lung lesion score for negative and positive animals tested using P22 ELISA with oral fluid samples.

In the present study, 22 out of the 64 TB-infected animals attained positive results to the P22 ELISA when using oral fluids, yielding an estimated Se of 34.4% (95% CI: 22.4–45.6). All the animals from the TB-free herd tested negative to the P22 ELISA when using oral fluid samples, attaining an Sp of 100% (95% CI: 96.7–100; Table 1). The use of the selected cut-off point made it possible to obtain the positive predictive value [100% (95% CI: 97.4–100)], negative predictive value [77.2% (95% CI: 71.1–83.2)], and area under the curve (0.827). Nineteen out of the 22 P22 ELISA-positive goats similarly had TB-compatible lesions in their lungs, and 20 out of 22 had them in different LNs (17 out of 22 in both locations). Taking into account only the setting of 16 animals with severe lesions (lesion score over 10), the ELISA showed an Se of 75% (95% CI: 53.7–96.2). Moreover, those animals that were positive to the P22 ELISA as regards oral fluid samples had a significantly (*p* = 0.018) higher lung TBL score (Figure 1).

**Table 1:** Results used to determine the sensitivity and specificity of the oral fluid P22 ELISA for diagnosis of tuberculosis in goats.

**Sample type**	**Presence of TB-lesions**	**Absence of TB-lesions**	**Total test results**
**Oral fluid ELISA test positive**	22^*^ (TP)	0 (FP)	22 (total test positives)
**Oral fluid ELISA test negative**	42 (FN)	113^**^ (TN)	184 (total test negatives)
**Total samples analyzed**	64	113	206 (total population)

* *Sensitivity of the P22 ELISA was 34.4% (22/64);*

** *Specificity of the P22 ELISA was 100% (113/113)*.

**Figure 2:**
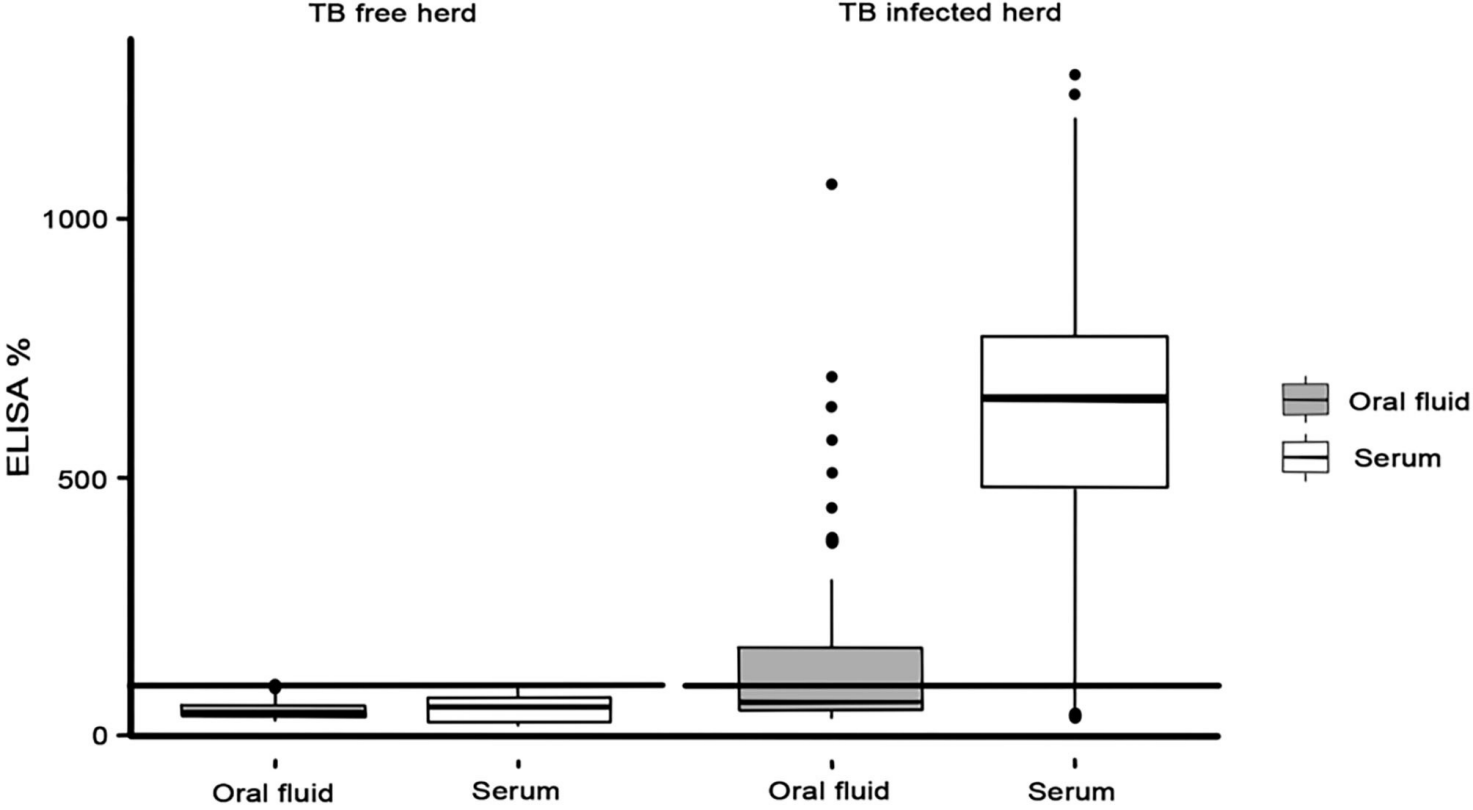
ELISA% in oral fluid (gray) and serum (white) samples observed in TB-free and TB-infected herds.

When using the serum samples, 56 out of the 64 *M. bovis*-infected goats were positive to the P22 ELISA (E% > 100), with an Se of 87.5% (77.2–93.5, 95% CI) (Table 2). The *M. bovis*-infected animals had a higher E% when employing the serum (median = 653.9 E%; *p* < 0.0001) than when employing the oral fluid samples (median = 66.6 E%) (Figure 2). Similar E% for oral fluid (median = 46.2 E%) and serum (median = 55.2 E%) samples were obtained from animals in the TB-free herd, and no significant differences were observed (*p* = 0.852). Finally, there was a very small correlation between the P22 results obtained for the oral fluid and serum samples (*R*^2^ = 0.032) and between the E% results obtained for the oral fluid and the TB compatible lesions observed in the lungs of the slaughtered goats (*R*^2^ = 0.142) and the head and pulmonary LNs: retropharyngeal (*R*^2^ = 0.206), tracheobronchial (*R*^2^ = 0.081), and mediastinal (*R*^2^ = 0.116) (Supplementary Material). The correlation between the oral fluid E% and the total lesion score was slightly higher (*R*^2^ = 0.225).

**Table 2:** Summary of the ante-mortem tuberculosis (TB) diagnostic tests and post-mortem analysis in the goats under study.

**Herd**	**Animals**	**SIT test^a^**	**CIT test^b^**	**IGRA^c^**	**P22 ELISA (serum)^d^**	**P22 ELISA (oral fluid)^d^**	**TB lesions in lungs**	**TB lesions in head and pulmonary lymph nodes**
***M. bovis*** **-infected**	64	55 (85.9%)	37 (57.8%)	38 (59.4%)	56 (87.5%)	22 (34.4%)	54 (84.4%)	60 (93.7%)
**TB-free**	113	0 (0%)	0 (0%)	0 (0%)	0 (0%)	0 (0%)	0 (0%)	0 (0%)

a *SIT, Single intradermal tuberculin test: protocol and interpretation was performed according to Council Directive 64/432/EEC and Royal Decree RD2611/1996*.

b *CIT, Comparative intradermal tuberculin test: protocol and interpretation was performed according to Council Directive 64/432/EEC and Royal Decree RD2611/1996*.

c *IGRA, interferon-gamma release assay: an animal was considered positive to IGRA if the optical density (OD) of a sample stimulated with bovine PPD minus the OD of the aliquot stimulated with PBS (nil) was >0.05 and greater than the OD of the sample stimulated with avian PPD*.

d *P22 ELISA, an animal was considered positive to ELISA p22 when the E% value was >150 (serum) or 100 (oral fluid)*.

Finally, with regard to the techniques based on cellular immune response, of the 64 goats in the TB-infected herd, 55 and 37 were considered positive reactors to the SIT test [(85.9% (95% CI: 75.3–92.4)] and the CIT test [(57.8% (95% CI: 45.6–69.1)], respectively. In this herd, 38/64 animals were positive to IGRA, with an apparent prevalence of 59.4% (95% CI: 47.1–70.5) (Table 2). In this respect, there was no correlation between IGRA and the total lesion score results (*R*^2^ = 0.004), which was lower than the correlation observed with E% when using oral fluid samples. A poor correlation was observed between SIT and CIT tests and the total lesion score results (*R*^2^ = 0.042 and 0.069, respectively) (Supplementary Material).

## Discussion

In the present study, an in-house ELISA with which to detect specific antibodies against *M. bovis* in oral fluid samples was developed and evaluated for the first time in goats. The results showed a high Sp but a limited Se of the P22 ELISA for TB diagnosis using oral fluid samples from goats.

The P22 ELISA had previously been evaluated for TB diagnosis in goats using sera and milk samples, showing promising results as regards Se and Sp (4, 8, 15). With regard to oral fluid samples for TB diagnosis, a previous study was carried out using a PPD-B-based ELISA in wild boar (13). However, to the best of the authors' knowledge, no previous studies using an ELISA for the detection of specific antibodies against TB in oral fluid samples from ruminants are available. With regard to Sp, the P22 ELISA achieved an excellent Sp of 100%, which was higher than that obtained by the serological tests described to date for the diagnosis of TB in goats using serum or milk samples (4, 7). Interference of vaccination against MAP on the diagnosis of TB in goats has been previously described in TB-free herds in other countries (30). Moreover, different studies have reported a lower Sp [58.3 (95% CI: 42.2–72.9)−96 (95% CI: 90.1–98.4)] of the P22 ELISA in MAP vaccinated goats than in non-vaccinated goats (4, 31). The higher Sp obtained in our study [100% (95% CI: 96.7–100)] using oral fluids was related to the low Se achieved. Therefore, although both the herds studied herein were vaccinated against *M. avium* subsp. *paratuberculosis* (MAP), a cross-reaction in the TB oral fluid test owing to MAP vaccination was not demonstrated in our study using the recommended cut-off point. However, despite the optimal performance of the P22 ELISA when using serum and milk samples, the present study showed that the usefulness of oral fluid samples as regards detecting *M. bovis* infection in goats was limited, unlike that which occurred in a previous study with wild boar that reported an Se of 67.3% (13). The performance of a diagnostic test is usually evaluated by taking into account the results of a bacteriological culture that is considered the gold standard. None of the *ante*-*mortem* tests used to define TB infection in goats are perfect in terms of Se and Sp, and they are not, therefore, accurate indicators of the real TB status of the animals. In this respect, the results of serology were used as a reference to define the infection status in the study of wild boar, and this could have affected the accuracy of the test (13). In fact, when the Se of the P22 ELISA carried out using oral fluid samples was estimated using serum results as a gold standard, the Se was slightly higher in our study [39.3% (95% CI: 27.6–52.4) data not shown], but still lower than that reported for wild boar.

It is necessary to state that in the present study, the P22 ELISA attained a higher Se when using serum samples than when using oral fluid samples. Moreover, the Se of TB diagnostic techniques based on specific-antibody detection can be improved by using samples collected after the PPD inoculation. This phenomenon, which is denominated as the booster effect, has been reported in different species (8, 32, 33). It has been suggested that the booster effect on the antibody response is a valuable methodology by which to increase the Se of serological assays in ruminants (6, 8). Further studies with which to evaluate this booster effect in oral fluid samples are, however, required.

The Se of the antibody detection tools is generally lower than that reported when using diagnostic tools based on cellular immune response (34). However, in this work, a higher Se was obtained when using serum samples (87.5%) than when using IGRA (59.4%), SIT (85.9%), and CIT (57.8%) tests, even in the absence of the booster effect. These results support the value of serological tests as a tool for TB diagnosis in ruminants, as observed in recent studies (4, 6, 8, 9), and the importance of developing new TB diagnostic tools in order to maximize the detection of infected animals.

It is widely known that the production and concentration in the samples of the different immunoglobulin (Ig) may differ between species. In this context, the IgG concentration in the oral fluid from goats is significantly lower than that in swine (35), which may explain the differences in the Se previously stated. The IgA-antibody levels in ruminants are, on the contrary, higher than in other species, which could explain the differences observed in the diagnosis when employing IgA or IgG based-ELISAs for other infections such as Schmallenberg disease (23). Moreover, recent studies support the theory that the detection of IgA in oral fluid samples appears to be more robust and stable over time in pigs (20). Ab production in oral fluid is related to mucosal immunity, and local antigen stimulation is, therefore, of paramount importance if high levels of IgA are to be attained in that sample. This could also explain the differences observed between serum and oral fluid samples. In this respect, in the present study, a better correlation between E% in oral fluid samples and the severity of lesions was observed in the retropharyngeal LNs. These LNs are closer to the salivary glands when compared to the pulmonary LNs (mediastinal and tracheobronchial), but additional studies are required in order to confirm the potential correlation between a more severe local immune response and a higher antibody production in ruminants.

Finally, it is necessary to state that, in this study, a goat model was used to evaluate the performance of the P22 ELISA when using oral fluid samples. Previous studies have shown promising results of antibody-based platforms for the diagnosis of other diseases (e.g., FMDV or SVB) when using oral fluid samples in cattle (22, 23). The results from the present study suggest a similar or limited performance of the P22 ELISA in oral fluid samples from cattle owing to the high diagnostic pressure as a consequence of the official eradication programmes, since it is difficult to find animals in an advance stage of infection. The realization of similar studies with which to confirm this hypothesis would, nevertheless, be interesting.

In conclusion, the use of oral fluid sample biomarkers for TB diagnosis in ruminants is still far from being routinely applied and requires further validation and research. The overall results obtained from the present study suggest that employing the P22 ELISA for the detection of specific antibodies in oral fluid samples is highly specific but has a limited value as regards detecting infected animals. Nevertheless, its performance is significantly higher in the presence of severe lesions, detecting a high proportion of those animals in the herd that have these lesions.

## Data Availability Statement

The original contributions presented in the study are included in the article/Supplementary Material, further inquiries can be directed to the corresponding author.

## Ethics Statement

Ethical review and approval was not required for the animal study because the animals in the present study were not considered as experimental animals. All handling and sampling procedures were performed in compliance with Spanish legislation (Royal Decree 720.7/2011). Written informed consent for participation was not obtained from the owners because the animals has an owner but he collaborates with our research center and he is aware of all the studies and publications (he prefers to be anonymous).

## Author Contributions

JO, JI-L, and JB wrote the manuscript and designed the figures. JO and JI-L performed the literature search. JO, JI-L, AG-B, and IA-R performed the experiments. JO, JI-L, JB, LJ, IM, IA-R, ÁR, LJ, BR, and MD interpreted the data. All the authors reviewed and approved the manuscript.

## Conflict of Interest

The authors declare that the research was conducted in the absence of any commercial or financial relationships that could be construed as a potential conflict of interest.

## Publisher's Note

All claims expressed in this article are solely those of the authors and do not necessarily represent those of their affiliated organizations, or those of the publisher, the editors and the reviewers. Any product that may be evaluated in this article, or claim that may be made by its manufacturer, is not guaranteed or endorsed by the publisher.
