# Supplementary figures and images for: Evaluation of P22 ELISA for the Detection of Mycobacterium bovis-Specific Antibody in the Oral Fluid of Goats
Source: Front Vet Sci. 2021 Aug 11;8:674636. doi: 10.3389/fvets.2021.674636 (PMC8385241; doi:10.3389/fvets.2021.674636)

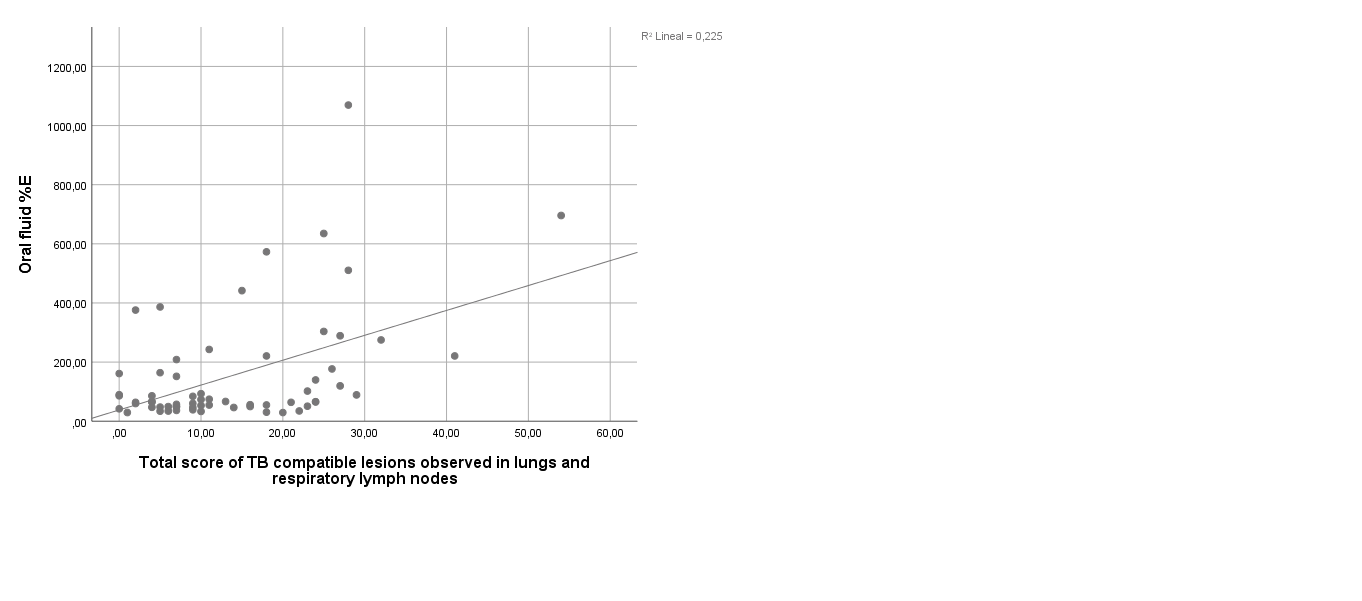


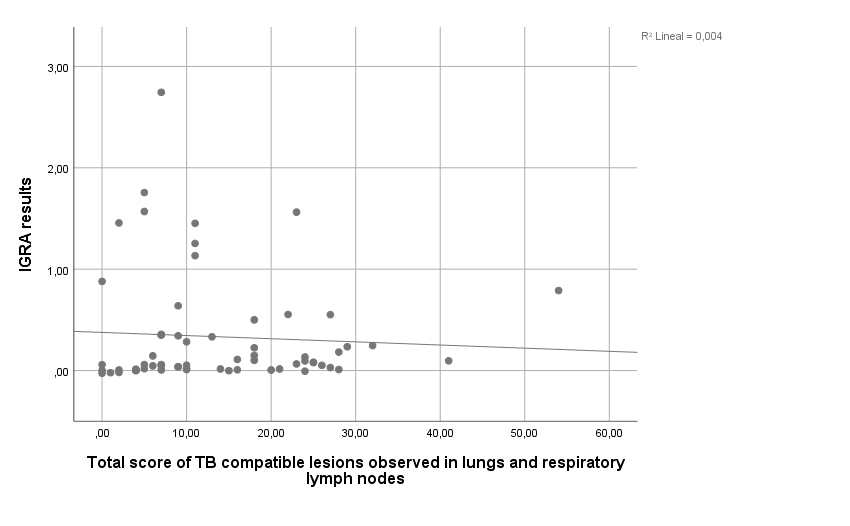


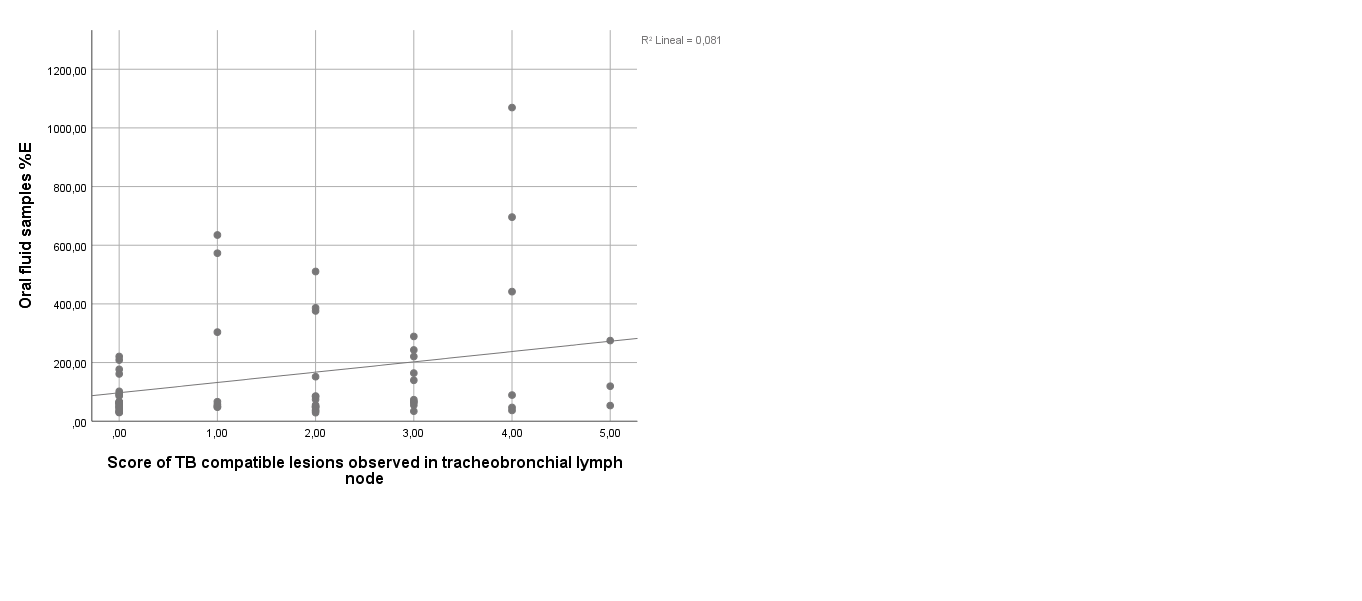


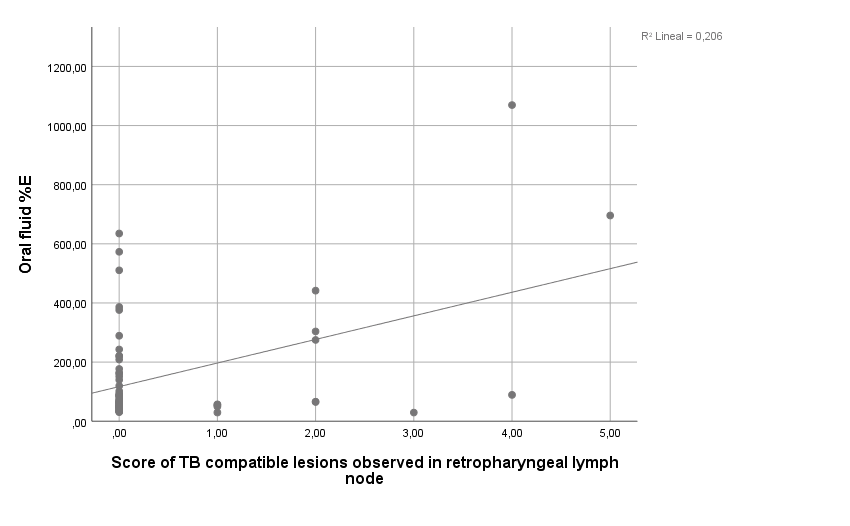


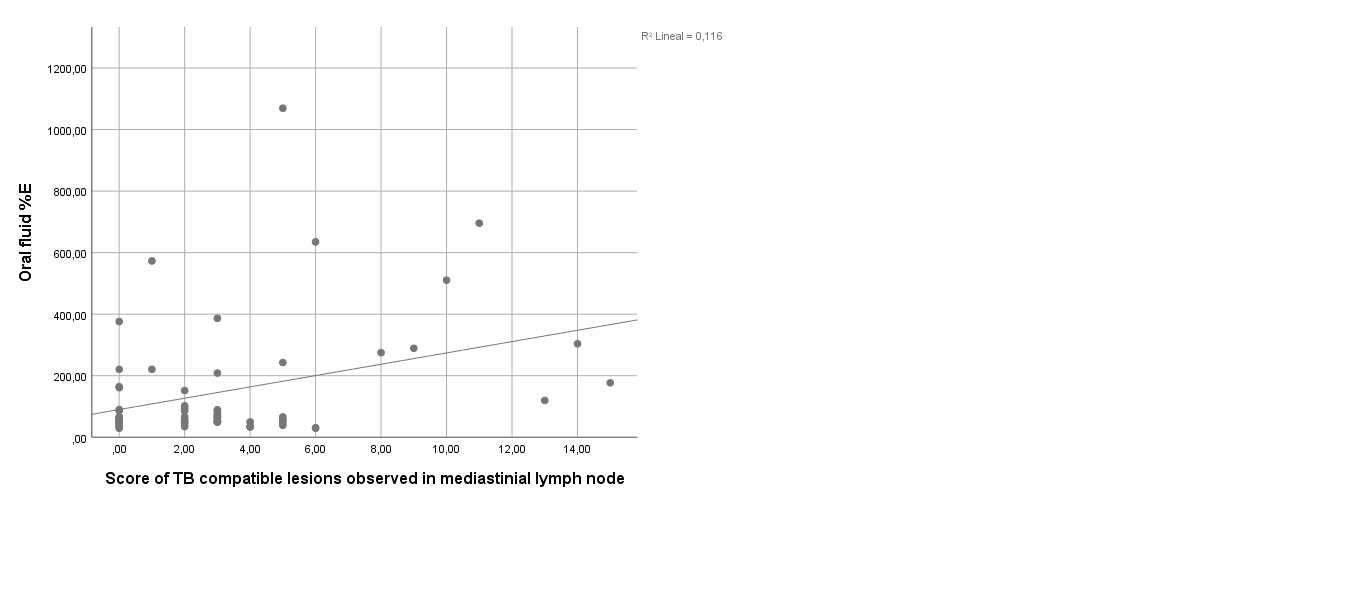


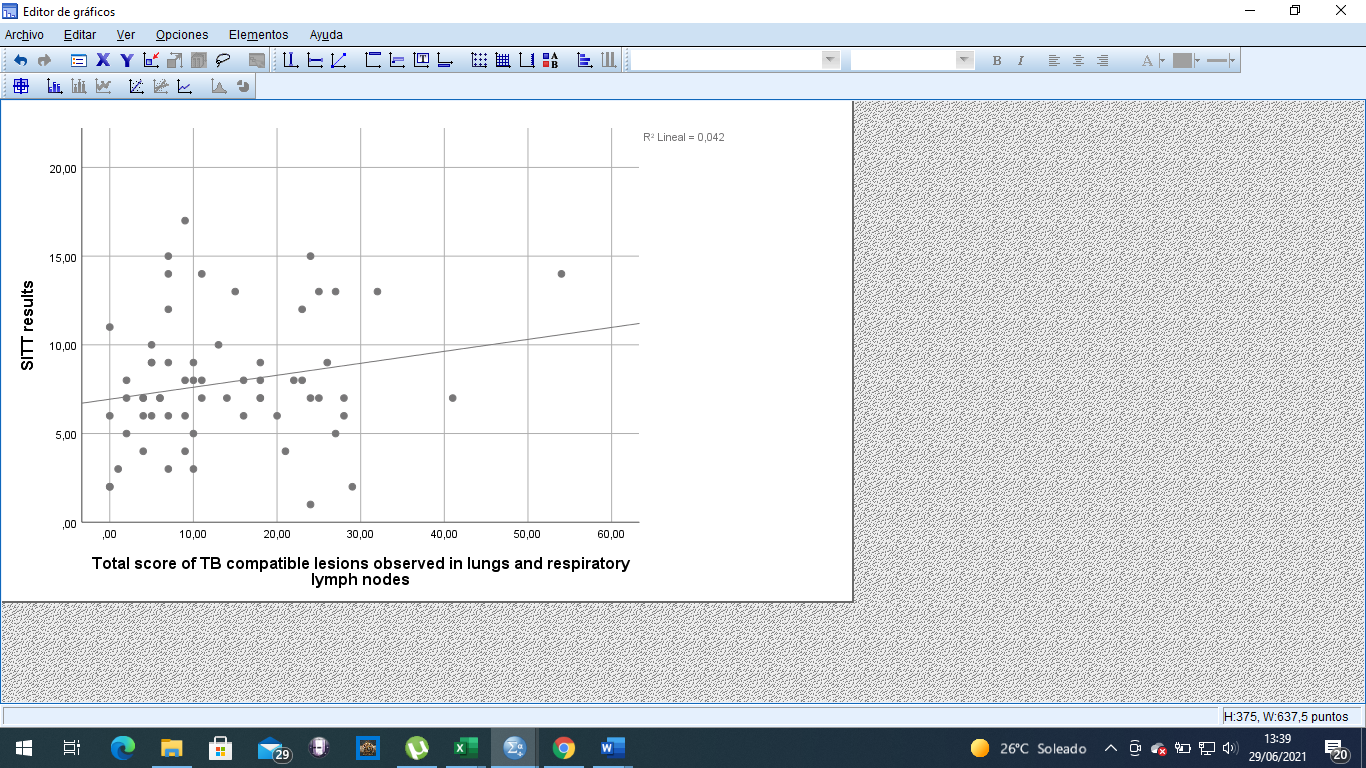


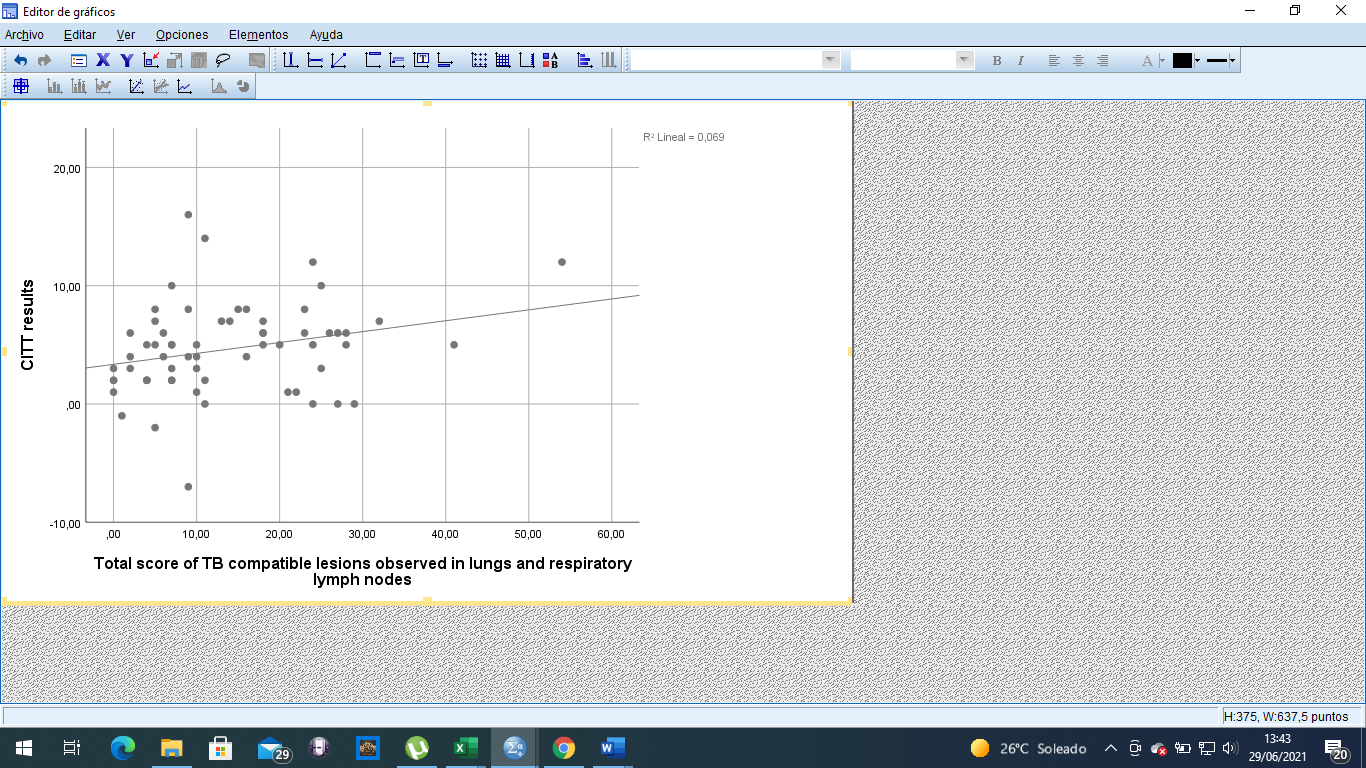


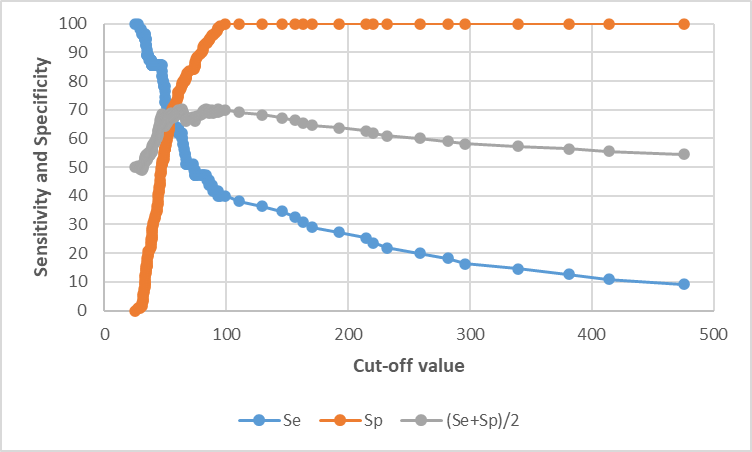


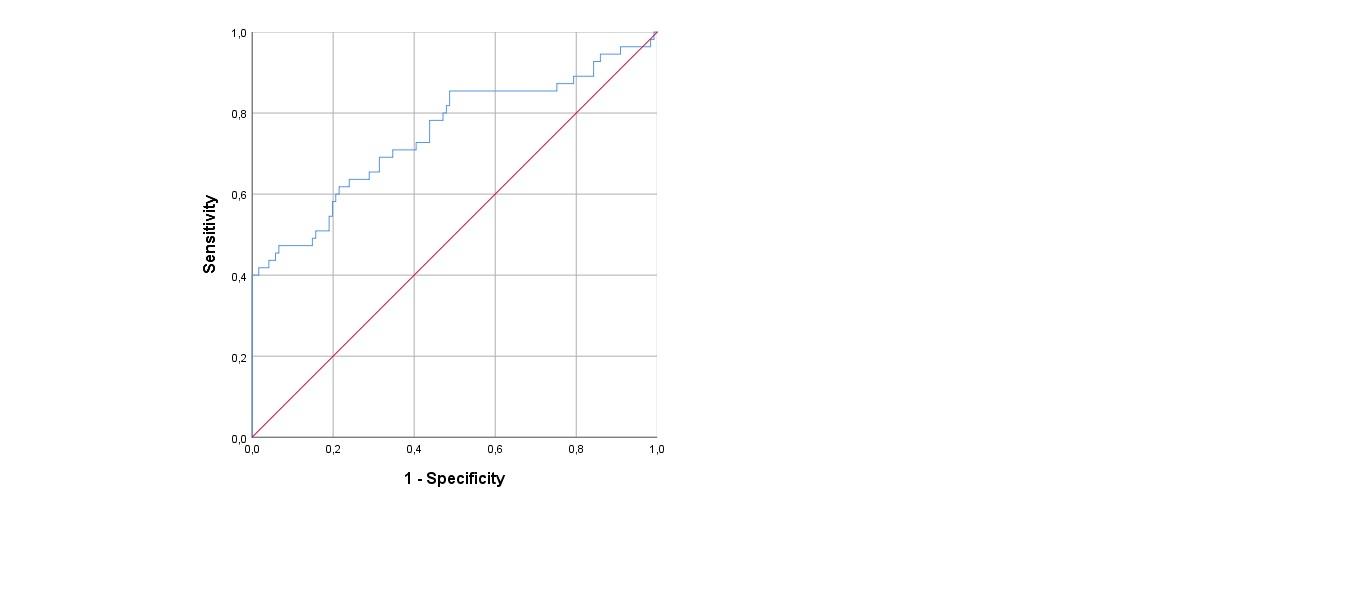

Supplement: Supplementary file 1 [file Data_Sheet_1.docx]
